# Supplementary material for: P-TRAP: a Panicle Trait Phenotyping tool
Source: BMC Plant Biol. 2013 Aug 29;13:122. doi: 10.1186/1471-2229-13-122 (PMC3848748; doi:10.1186/1471-2229-13-122)
Supplement: Additional file 7 — P-TRAP source code. The Netbeans project that contains the source code of the application. [file 1471-2229-13-122-S7.zip › javadoc.zip!/javadoc/org-biolag-particleprocessor/org/biolag/particleprocessor/Moments.html]

Moments (ParticleProcessor)


JavaScript is disabled on your browser.


- Package
- Class
- Tree
- Deprecated
- Index
- Help

*org.biolag.particleprocessor 1.0*

- Prev Class
- Next Class

- Frames
- No Frames

- All Classes

- Summary:
- Nested |
- Field |
- Constr |
- Method

- Detail:
- Field |
- Constr |
- Method


org.biolag.particleprocessor

## Class Moments

- java.lang.Object
- - org.biolag.particleprocessor.Moments

- ---

    

  ```
  public abstract class Moments
  extends java.lang.Object
  ```

  The moments manipulator class.

  Author:
  :   faroq

- - ### Constructor Summary

    Constructors

    Constructor and Description

    `Moments()`
  - ### Method Summary

    Methods

    | Modifier and Type | Method and Description |
    | --- | --- |
    | `static double` | `centeralMoment(int p, int q, java.util.ArrayList<java.awt.Point> ROI)` Calculate the central moments |
    | `static double` | `compactness(java.util.ArrayList<java.awt.Point> ROI)` Calculate the compactness of set of of points. |
    | `static double` | `ellipticity(java.util.ArrayList<java.awt.Point> ROI)` Calculate the ellipticity of a set of points. |
    | `static double` | `moment(int p, int q, java.util.ArrayList<java.awt.Point> ROI)` |
    | `static double` | `nomalizedCenteralMoment(int p, int q, java.util.ArrayList<java.awt.Point> ROI)` A normalized version of `centeralMoment(int, int, java.util.ArrayList)` function |
    | `static double` | `orientation(java.util.ArrayList<java.awt.Point> ROI)` Calculate the orientation angle of a set of points |
    | `static java.util.ArrayList<java.lang.Double>` | `widthANDheight_bruteforce(java.util.ArrayList<java.awt.Point> ROI, java.util.ArrayList<java.awt.Point> CROI, java.util.ArrayList<java.awt.Point> out_MajoranfMinorAxes_corrdinates)` Calculate the width and height of the particles by examining all points and finding the major and minor axes. |
    | `static java.util.ArrayList<java.lang.Double>` | `widthANDheight(java.util.ArrayList<java.awt.Point> ROI, java.util.ArrayList<java.awt.Point> CROI, java.util.ArrayList<java.awt.Point> out_MajoranfMinorAxes_corrdinates)` Calculate the width and height of a set of points |

    - ### Methods inherited from class java.lang.Object

      `clone, equals, finalize, getClass, hashCode, notify, notifyAll, toString, wait, wait, wait`

- - ### Constructor Detail


    - #### Moments

      ```
      public Moments()
      ```
  - ### Method Detail


    - #### moment

      ```
      public static double moment(int p,
                  int q,
                  java.util.ArrayList<java.awt.Point> ROI)
      ```


    - #### centeralMoment

      ```
      public static double centeralMoment(int p,
                          int q,
                          java.util.ArrayList<java.awt.Point> ROI)
      ```

      Calculate the central moments

      Parameters:
      :   `p` - the degree of the first part
      :   `q` - the degree of the second part
      :   `ROI` - the points

      Returns:
      :   a double value


    - #### nomalizedCenteralMoment

      ```
      public static double nomalizedCenteralMoment(int p,
                                   int q,
                                   java.util.ArrayList<java.awt.Point> ROI)
      ```

      A normalized version of `centeralMoment(int, int, java.util.ArrayList)` function

      Parameters:
      :   `p` -
      :   `q` -
      :   `ROI` -

      Returns:
      :   double value


    - #### orientation

      ```
      public static double orientation(java.util.ArrayList<java.awt.Point> ROI)
      ```

      Calculate the orientation angle of a set of points

      Parameters:
      :   `ROI` - the list of points

      Returns:
      :   a double valued angle


    - #### compactness

      ```
      public static double compactness(java.util.ArrayList<java.awt.Point> ROI)
      ```

      Calculate the compactness of set of of points.

      Parameters:
      :   `ROI` - the list of points.

      Returns:


    - #### widthANDheight

      ```
      public static java.util.ArrayList<java.lang.Double> widthANDheight(java.util.ArrayList<java.awt.Point> ROI,
                                                         java.util.ArrayList<java.awt.Point> CROI,
                                                         java.util.ArrayList<java.awt.Point> out_MajoranfMinorAxes_corrdinates)
      ```

      Calculate the width and height of a set of points

      Parameters:
      :   `ROI` - the list of points
      :   `CROI` - the contour of the ROI points
      :   `out_MajoranfMinorAxes_corrdinates` - a list of points of the major
          axes- this is an out param.

      Returns:
      :   the width and height in a list of two doubles.


    - #### widthANDheight\_bruteforce

      ```
      public static java.util.ArrayList<java.lang.Double> widthANDheight_bruteforce(java.util.ArrayList<java.awt.Point> ROI,
                                                                    java.util.ArrayList<java.awt.Point> CROI,
                                                                    java.util.ArrayList<java.awt.Point> out_MajoranfMinorAxes_corrdinates)
      ```

      Calculate the width and height of the particles by examining all points
      and finding the major and minor axes.

      Parameters:
      :   `ROI` - the list of points
      :   `CROI` - the contour of the list of points
      :   `out_MajoranfMinorAxes_corrdinates` -

      See Also:
      :   `widthANDheight(java.util.ArrayList, java.util.ArrayList,
          java.util.ArrayList)`


    - #### ellipticity

      ```
      public static double ellipticity(java.util.ArrayList<java.awt.Point> ROI)
      ```

      Calculate the ellipticity of a set of points.

      Parameters:
      :   `ROI` - the point list

      Returns:
      :   a double value


- Package
- Class
- Tree
- Deprecated
- Index
- Help

*org.biolag.particleprocessor 1.0*

- Prev Class
- Next Class

- Frames
- No Frames

- All Classes

- Summary:
- Nested |
- Field |
- Constr |
- Method

- Detail:
- Field |
- Constr |
- Method
